# Finite element analysis of PFNA in the treatment of osteoporotic intertrochanteric A2.3 fractures of the femur

**DOI:** 10.3389/fbioe.2026.1887294

**Published:** 2026-07-22

**Authors:** Fan Wang, Na Liu, Ruohan Pan, Yanhui Zhang, Chengdong Chen, Chaochao Huang, Jian Wang, Haihan Song, Xingpeng Zhang, Wen Wang

**Affiliations:** 1 Shandong First Medical University and Shandong Academy of Medical Sciences, Jinan, Shandong, China; 2 Tianjin Walkman Biomaterial Co., Ltd., Newton Laboratory, Tianjin, China; 3 Department of Orthopaedics, The First Affiliated Hospital of Shandong First Medical University and Shandong Provincial Qianfoshan Hospital, Jinan, Shandong, China; 4 Department of Orthopedics, Shanghai Pudong New Area People’s Hospital, Shanghai, China

**Keywords:** finite element analysis, intertrochanteric femoral fracture, intramedullary nail length, osteoporosis, PFNA

## Abstract

**Background:**

Poor bone quality in osteoporosis can compromise implant fixation and stability in intertrochanteric fractures. This study employed finite element analysis to evaluate the biomechanical performance of short (170 mm), medium (240 mm), and long (320 mm) proximal femoral nail antirotation (PFNA) implants in osteoporotic intertrochanteric A2.3 fractures. By modulating the elastic modulus of cancellous bone to simulate varying degrees of osteoporosis severity, we aimed to identify the optimal PFNA length for clinical practice.

**Methods:**

A three-dimensional finite-element model of an osteoporotic intertrochanteric A2.3 femoral fracture was established. The elastic modulus of cancellous bone was assigned six levels (588, 445, 260, 115, 63, and 34 MPa), representing different osteoporosis severities. PFNA implants of 170, 240, and 320 mm lengths were assembled, with the 170 mm nail further evaluated at diameters of 9.5, 10, and 11 mm. Single-leg stance loading was simulated. Von Mises peak stresses in the PFNA implant, cortical bone, and cancellous bone, along with maximum displacement, were calculated and compared.

**Results:**

The 240 mm PFNA exhibited the lowest von Mises peak stress (442.86–560.44 MPa) across all bone quality conditions. The 170 mm PFNA exceeded 1000 MPa when bone quality was ≤260 MPa, surpassing the yield strength of titanium alloy (approximately 900 MPa). The 320 mm PFNA demonstrated intermediate stress (618.60–842.16 MPa). The 240 mm PFNA consistently produced the lowest cortical stress (85.191–91.948 MPa) and similar femoral displacement to the 320 mm PFNA, while cancellous bone stress decreased progressively with deteriorating bone quality (19.68→8.35 MPa). The 170 mm PFNA showed higher cortical stress (185.25–211.7 MPa) and the greatest displacement (8.05–9.44 mm), whereas the 320 mm PFNA exhibited a sharp increase when bone quality was ≤115 MPa, reaching 472.82 MPa at 34 MPa. For the 170 mm nail, increasing diameter from 9.5 mm to 11 mm reduced peak implant stress by 25.8%–49.0%, with the 11 mm nail remaining below the yield threshold in all but the most severe osteoporotic condition.

**Conclusion:**

Within the constraints of the present finite element model, the 240 mm PFNA showed the most favorable overall comparative biomechanical performance among the tested configurations and may represent a reasonable biomechanical option for osteoporotic intertrochanteric A2.3 fractures.

## 1 Introduction

Osteoporosis is a systemic skeletal disease characterized by low bone mass and microarchitectural deterioration of bone tissue, with a consequent increase in bone fragility and susceptibility to fracture (NIH Consensus Development Panel on Osteoporosis Prevention, 2001). Intertrochanteric femoral fractures are common and severe among elderly patients with osteoporosis, and the compromised bone quality increases the risk of implant failure, cutout, and varus deformity (Zhu et al., 2015; Wang et al., 2023). According to the AO/OTA classification, 31-A2.3 fractures are unstable multifragmentary intertrochanteric fractures, typically associated with comminution and compromised medial support (Meinberg et al., 2018).

Proximal femoral nail antirotation (PFNA) is widely used for unstable intertrochanteric fractures, particularly in elderly patients with osteoporotic bone, owing to its intramedullary design and favorable rotational stability (Wang et al., 2023; Horwitz et al., 2016; Yu et al., 2016). However, the optimal PFNA length selection remains controversial. Although longer nails (e.g., 320 mm) theoretically offer enhanced stability through increased lever arms, they entail greater surgical trauma and raise concerns regarding iatrogenic fractures after implantation (Horwitz et al., 2016; Shannon et al., 2019). Short nails (e.g., 170 mm) offer simplified insertion, yet their limited anchorage length may make it difficult to counteract substantial stresses at the fracture site in the osteoporotic bone, potentially resulting in inadequate stability (Horwitz et al., 2016). Medium-length nails (e.g., 240 mm) may represent a compromise between these approaches; however, systematic evidence confirming whether their biomechanical performance in osteoporotic models can match that of long nails remains limited.

Previous finite element studies evaluating nail length in proximal femoral fracture fixation have largely relied on single-bone quality assumptions (Je et al., 2024; Hong et al., 2017; Kwak et al., 2021),making it difficult to comprehensively assess their applicability across osteoporotic patient populations. By modulating the elastic modulus of cancellous bone to simulate varying degrees of osteoporosis severity, this study established a three-dimensional (3D) finite element model of an osteoporotic intertrochanteric A2.3 femoral fracture. We systematically compared the biomechanical effects of three PFNA lengths, focusing on stress and strain distribution in both the implants and bone tissue under osteoporotic conditions, thereby providing evidence-based guidance for PFNA length selection in osteoporotic intertrochanteric A2.3 fractures from a biomechanical perspective.

## 2 Materials and methods

### 2.1 Establishment of the three-dimensional finite element model

**FIGURE 1:**
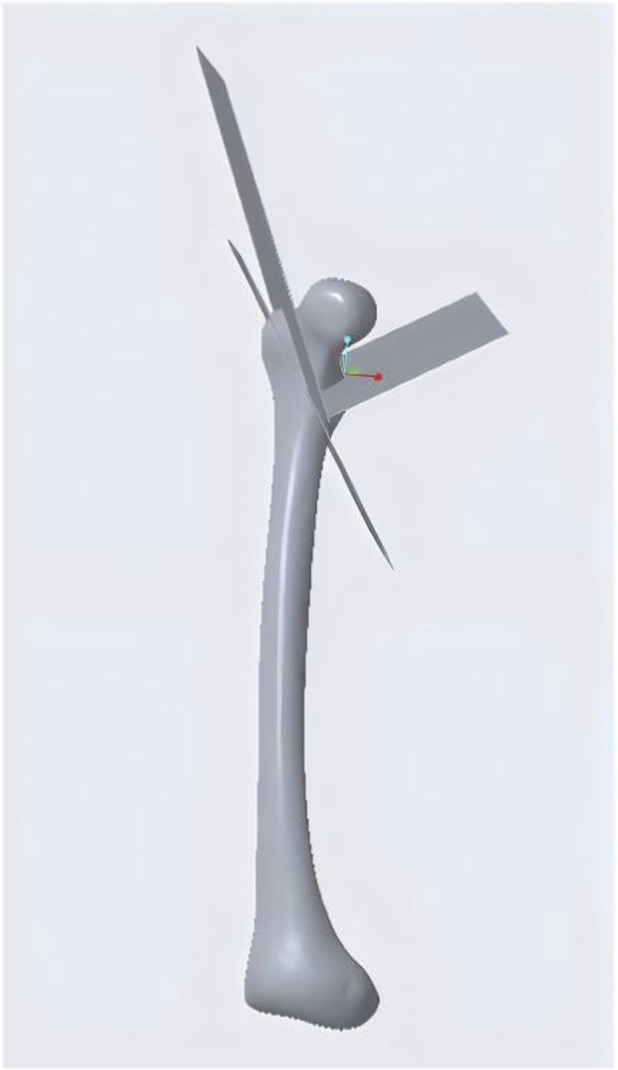
Construction of the A2.3 intertrochanteric fracture model using three intersecting planes. The first plane is positioned 10 mm below the greater trochanter at an angle of 20° to the femoral shaft axis; the second plane is tangential to the superior margin of the lesser trochanter; the third plane connects the intersection line of the first two planes to the apex of the greater trochanter. The intersection of these three planes with the femur defines the fracture fragments according to the AO/OTA A2.3 classification.

**FIGURE 2:**
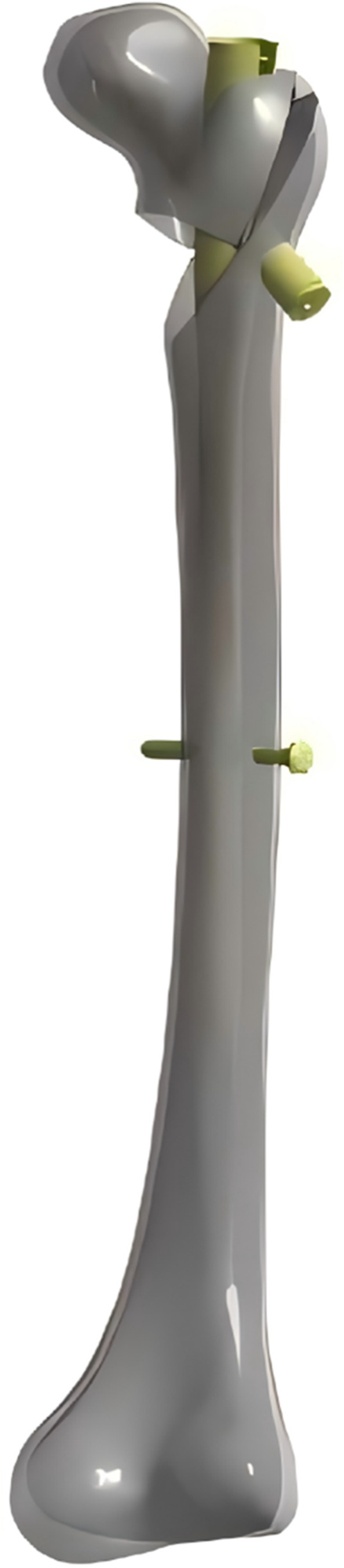
Finite element model of the femur with PFNA implant for A2.3 intertrochanteric fracture. The model includes the cortical bone (semi-transparent), cancellous bone, and the PFNA implant consisting of the main nail, spiral blade, and distal locking screw. The A2.3 intertrochanteric fracture fragments are visible.

This study used the structural and morphological characteristics of a previously validated three-dimensional finite element femur model (Li et al., 2019a; Cha et al., 2024). The geometric shape of the 3D femur was derived from CT image data of an 86-year-old postmenopausal woman (60 kg, 165 cm). Individual patient characteristics were not considered for bone properties; instead, the osteoporotic bone characteristics reported in a previous study (Je et al., 2024) were adopted. The data were imported into medical image processing software (Mimics 21.0, Materialise, Belgium) for 3D reconstruction, to generate the femoral geometric model. The cortical and trabecular bones of the femur were processed using the GEOMAGIC Studio v2013 software (3D Systems, Rock Hill, SC, USA) during the point, polygon, and shape stages to generate models of the femoral cortical and trabecular bone. These bone models were imported and assembled. Subsequently, PFNA implants of varying lengths and other internal fixation components were inserted into the 3D model using Creo 2.0 (Parametric). Concurrently, an A2.3 fracture model was created according to the AO classification principles. The specific modeling method involved creating fracture lines using three intersecting planes. The first fracture line was positioned 10 mm below the greater trochanter, subsequently at an angle of 20° relative to the femoral shaft axis (Nie et al., 2022; Li et al., 2019b). The second fracture line was tangential to the superior margin of the lesser trochanter, while the third fracture line connected the intersection of the first and second lines to the apex of the greater trochanter (Figure 1). The processed model was imported into the ANSYS WorkBench 19 software, where the slicing function was used to construct the fracture model (Figure 2). The global element size was set to 2 mm. For the representative 240 mm PFNA model, the final mesh comprised approximately 293,832 elements and 454,253 nodes. Similar mesh densities were used for the other implanted models. Mesh quality was verified using the element quality metric in ANSYS WorkBench (Average 0.824, SD 0.118), indicating satisfactory quality.

### 2.2 Material properties

For model standardization and between-group comparability, both cortical and cancellous bone were modeled using simplified linear elastic material properties, as commonly adopted in previous finite element studies (Long et al., 2024; Jiang et al., 2022; Mahapatra and Pal, 2024; Li et al., 2019c). To overcome the limitations of a single-patient sample and to simulate patients with varying degrees of osteoporosis severity, this study treated the elastic modulus of the cancellous bone as a variable. Material properties for the osteoporotic femur and PFNA implant were assigned on the basis of previously published finite element studies (Kwak et al., 2021; Linde and Hvid, 1989; Stoffel et al., 2003), with the implant defined as Ti6Al4V titanium alloy. To simulate relative differences in osteoporotic bone deterioration, the elastic modulus of cancellous bone was reduced stepwise while maintaining the same geometric model. This approach was based on previous finite element studies and on the established relationship between trabecular bone density and elastic modulus, whereby reduced bone density is associated with lower mechanical stiffness (Kwak et al., 2021; Cha et al., 2024; Morgan et al., 2003; Helgason et al., 2008). The upper-limit elastic modulus (588 MPa) was adopted from published data on osteoporotic femoral trabecular bone (Cha et al., 2024), whereas the lower-limit value (34 MPa) was derived from a previously reported osteoporotic numerical model (Kwak et al., 2021). Four additional intermediate values (445, 260, 115, and 63 MPa) were generated along a logarithmic gradient between these two limits, resulting in six cancellous bone material-property sets. These values were intended to represent relative differences in bone quality rather than direct one-to-one correspondence with patient-specific DXA-derived T-scores. For cancellous bone, Poisson’s ratio was uniformly set to 0.20 across all six elastic modulus groups, as this value was considered a reasonable constant approximation for the simulated range of bone quality conditions and enabled standardized comparison among groups. Cortical bone properties remained fixed (E = 17,000 MPa, ν = 0.3). Three PFNA lengths (170, 240, and 320 mm) were combined with the six bone quality configurations to establish 18 finite element models, comprehensively evaluating the PFNA biomechanical performance under varying bone quality conditions. All three PFNA implants were sourced from Tianjin Weiman Biomaterial Co., Ltd., China, and a single distal locking screw was used for all nail lengths to standardize the fixation construct and enable controlled comparison of the biomechanical effect of nail length. Bone groups A-F correspond to elastic moduli of 588, 445, 260, 115, 63, and 34 MPa, respectively. The material properties used in the finite element models are summarized in Table 1.

**TABLE 1:** Material properties assigned in the finite element models.

Property	Cortical bone	Cancellous bone (Osteoporosis)						Implant (Ti6Al4V Titanium Alloy)
Elastic modulus, E (MPa)	17000	588	445	260	115	63	34	113800
Poisson’s ratio	0.3	0.2	0.2	0.2	0.2	0.2	0.2	0.342

Bone quality groups A–F correspond to cancellous bone elastic moduli of 588, 445, 260, 115, 63, and 34 MPa, respectively.

Ethics approval: This study was approved by the Medical Ethics Committee of the First Affiliated Hospital of Shandong First Medical University (Shandong Provincial Qianfoshan Hospital) (Approval No. [2026] Lun Shen Zi (S601)). The study used CT data from a single patient and was conducted in accordance with the ethical standards of the 1964 Declaration of Helsinki. Written informed consent was obtained from the patient for the use of the CT data for research purposes.

### 2.3 Loads and boundary conditions

All degrees of freedom at the distal femur were constrained (Jiang et al., 2022; Mahapatra and Pal, 2024; Li et al., 2019c; Mahapatra and Pal, 2025). A single-leg stance was assumed to simulate normal walking. Hip joint forces applied to the femoral head (1800 N, 300% of body weight) and abductor forces (600 N, 100% of body weight) were applied to the lateral surface of the greater trochanter (Taylor, 2006). Guided by the literature (Li et al., 2019a), the femoral head load was applied at 10° to the vertical axis in the coronal plane and 9° to the vertical axis in the sagittal plane. To standardize intergroup comparisons and represent an idealized immediate postoperative fixation state, all implant-bone interfaces and implant-component contacts were defined as bonded, representing an idealized fixation state without interfacial sliding or separation. Similar fully constrained interface assumptions, including bonded or tie contact definitions, have been adopted in previous finite element studies of proximal femoral fixation (Kwak et al., 2021; Long et al., 2024). Fracture surfaces were also modeled without relative sliding. This simplification may overestimate construct stability and was considered in the interpretation of the results. Because bonded contact precludes interfacial sliding and separation, no frictional contact parameters were assigned in the present model.

### 2.4 Experimental grouping and outcome measures

To simulate patients with varying degrees of osteoporosis severity, the elastic modulus of cancellous bone was set at six levels (588, 445, 260, 115, 63, and 34 MPa). These six bone states were combined with three PFNA lengths (170, 240, and 320 mm) to establish 18 three-dimensional finite element models. For ease of analysis, the six bone quality levels were categorized into severity as mild (588 and 445 MPa, corresponding to groups A and B), moderate (260 and 115 MPa, corresponding to groups C and D), and severe (63 and 34 MPa, corresponding to groups E and F). Based on the literature (Horwitz et al., 2016), the experiments were grouped into three categories based on the PFNA length: Group I (170 mm PFNA), Group II (240 mm PFNA), and Group III (320 mm PFNA). Each group had mild, moderate, and severe bone quality conditions. Additionally, to evaluate the effect of nail diameter on the mechanical performance of the 170 mm short nail, three diameters (9.5, 10, and 11 mm) were modeled under identical bone quality and loading conditions. The following mechanical metrics were evaluated:

1. Von Mises peak stress of the PFNA under different bone quality conditions;
2. Von Mises peak stress in osteoporotic cortical bone and each cortical bone fracture fragment under different bone quality conditions;
3. Von Mises peak stress in osteoporotic cancellous bone and each cancellous bone fracture fragment under different bone quality conditions;
4. Maximum femoral displacement of the osteoporotic fracture-implant composite model under different bone quality conditions;
5. Von Mises peak stress in 170 mm PFNA implants with different diameters (9.5, 10, and 11 mm) under varying bone quality conditions.

All metrics were calculated under identical loading and boundary conditions to enable comparison of biomechanical performance across different bone quality, nail length, and nail diameter combinations.

## 3 Results

### 3.1 PFNA stress analysis

**FIGURE 3:**
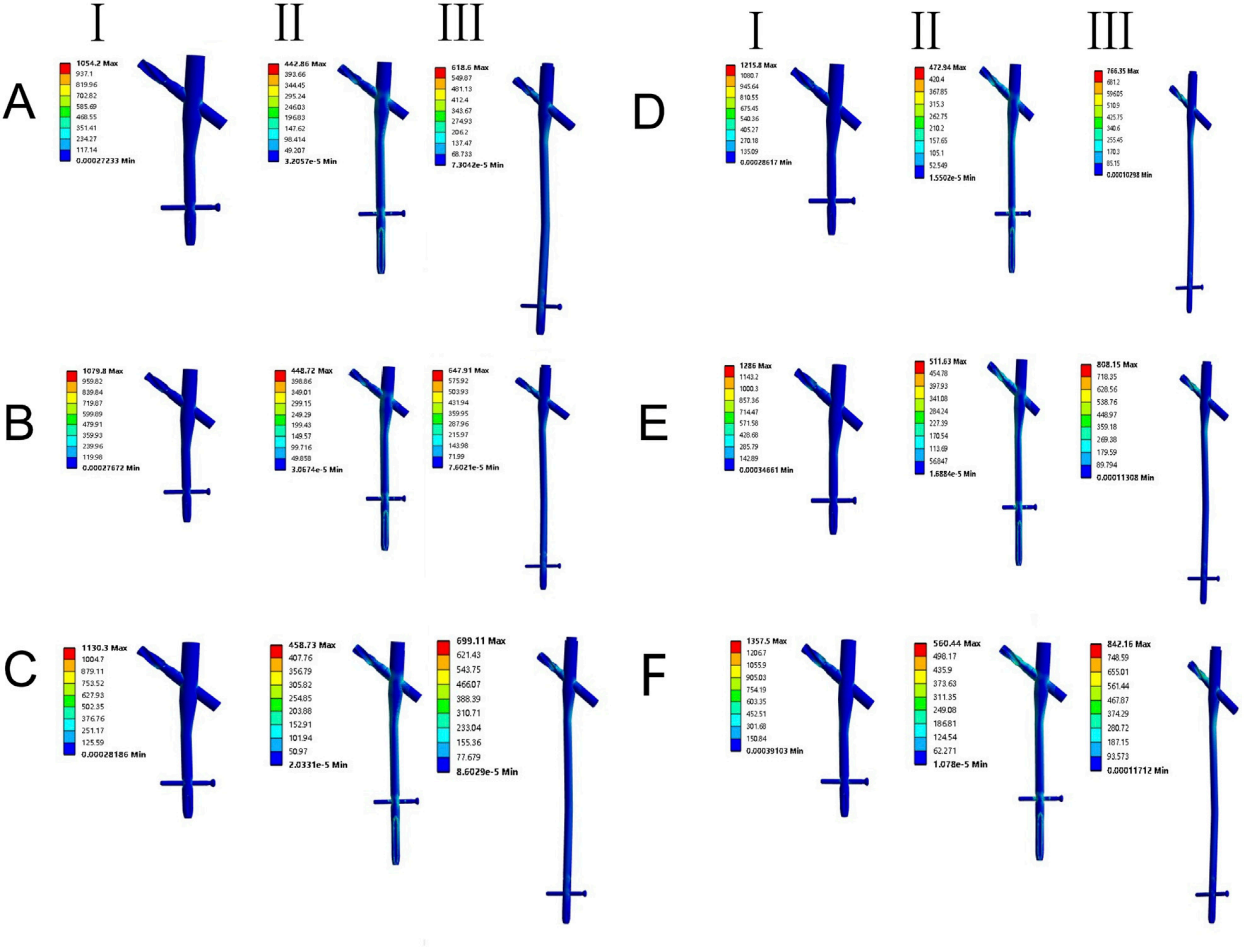
Von Mises stress distribution in PFNA implants under six bone quality conditions. Rows correspond to bone groups **(A–F)**, representing cancellous bone elastic moduli of 588, 445, 260, 115, 63, and 34 MPa, respectively. Columns correspond to PFNA lengths of 170 mm (I), 240 mm (II), and 320 mm (III). Stress values are expressed in MPa, with blue indicating lower stress and red indicating higher stress. Stress concentration was mainly observed at the junctions between the spiral blade and the main nail and between the distal locking screw and the main nail. For grayscale printing, readers are advised to use the numerical labels (Min to Max) provided along the color bar for accurate value quantification.

The von Mises stress distribution contour plots of the PFNA implants (Figure 3) showed that stress concentration primarily occurred at the spiral blade-main nail junction and distal locking screw-main nail junction. The 170 mm short nail demonstrated maximum peak stresses that ranged from 1054.20 to 1357.50 MPa across the six bone quality conditions, and increased as bone quality deteriorated. When bone quality decreased to 260 MPa, the peak stress reached 1130.30 MPa, exceeding the reported nominal yield strength of Ti6Al4V alloy (approximately 900 MPa), suggesting a higher relative risk of mechanical overload under the simulated loading condition. Stress concentration consistently occurred at the distal locking screw-main nail junction. The 240 mm medium-length nail exhibited maximum peak stresses of 442.86–560.44 MPa across all six bone quality conditions—the lowest among the three groups—with stress concentration at the spiral blade-main nail junction. The 320 mm long nail demonstrated maximum peak stresses ranging from 618.60 to 842.16 MPa across the six bone quality conditions, with stress concentration occurring similarly at the spiral blade-main nail junction. It should be noted that the nominal yield strength of Ti6Al4V alloy does not directly represent the clinically allowable stress threshold of the implant, as fatigue loading, factor of safety considerations, and other *in vivo* service conditions may substantially reduce the practical mechanical limit. Accordingly, the von Mises stress values reported in this study should be interpreted as comparative biomechanical indicators among constructs rather than absolute predictors of clinical safety or implant failure. The peak von Mises stress of PFNA implants of different lengths under varying bone quality conditions is presented in Table 2.

**TABLE 2:** Peak von Mises stress in PFNA implants of different lengths under different bone-quality conditions.

Bone grouping	Elastic modulus (MPa)	PFNA length	Peak stress under internal fixation (MPa)
A	588	170 mm	1054.20
		240 mm	442.86
		320 mm	618.60
B	445	170 mm	1079.80
		240 mm	448.72
		320 mm	647.91
C	260	170 mm	1130.30
		240 mm	458.73
		320 mm	699.11
D	115	170 mm	1215.80
		240 mm	472.94
		320 mm	766.35
E	63	170 mm	1286.00
		240 mm	511.63
		320 mm	808.15
F	34	170 mm	1357.50
		240 mm	560.44
		320 mm	842.16

Bone groups A–F represent cancellous bone elastic moduli of 588, 445, 260, 115, 63, and 34 MPa, respectively. PFNA, proximal femoral nail antirotation.

### 3.2 Cortical bone stress analysis

**FIGURE 4:**
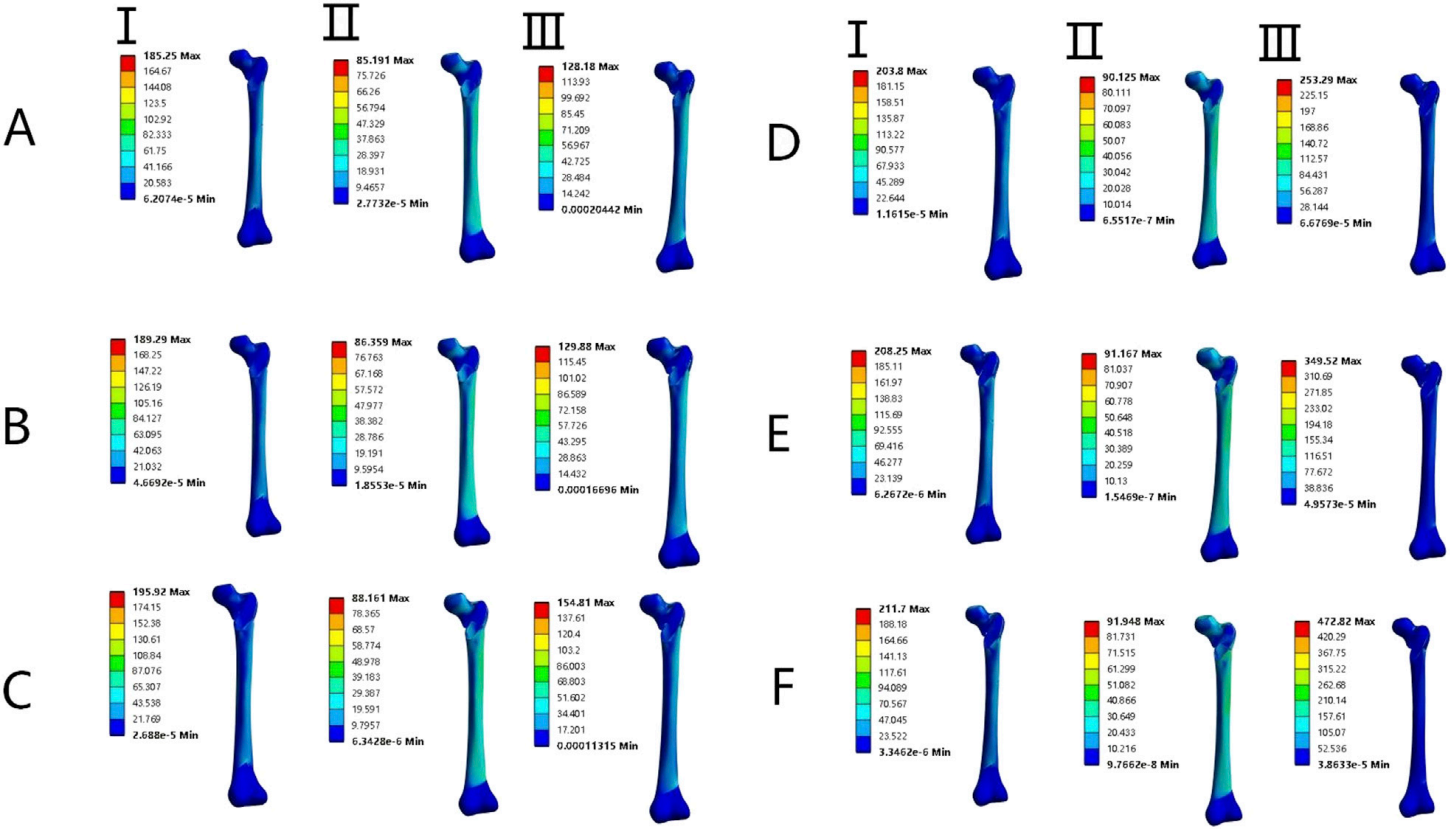
Von Mises stress distribution in cortical bone under six bone quality conditions with different PFNA lengths. Rows correspond to bone groups **(A–F)**, representing cancellous bone elastic moduli of 588, 445, 260, 115, 63, and 34 MPa, respectively. Columns correspond to PFNA lengths of 170 mm (I), 240 mm (II), and 320 mm (III). Stress values are expressed in MPa, with blue indicating lower stress and red indicating higher stress. Peak cortical stress was mainly located in the distal femur. For grayscale printing, readers are advised to use the numerical labels (Min to Max) provided along the color bar for accurate value quantification.

The von Mises stress distribution contour plots for the cortical bone (Figure 4) indicated that all three intramedullary nail groups exhibited maximum stress at the distal femur. The 170 mm short nail demonstrated peak cortical stresses that ranged from 185.25 to 211.70 MPa across all six bone quality conditions, and consistently maintained elevated values. Such localized high stress readily induces microdamage or pain in the distal cortical bones of patients with osteoporosis. The 240 mm medium-length nail exhibited peak cortical stresses of 85.191–91.948 MPa across all six bone quality conditions, the lowest among the three groups, with minimal variation across bone types, demonstrating a significant protective advantage. The 320 mm long nail showed peak cortical stresses ranging from 128.18 to 472.82 MPa across the six bone quality conditions. Stress rose sharply when bone strength fell below 115 MPa, reaching 472.82 MPa at 34 MPa—more than five times that of the 240 mm nail. The peak von Mises stress in cortical bone of PFNA fracture models of different lengths under various bone quality conditions is presented in Table 3.

**TABLE 3:** Peak von Mises stress in cortical bone under different bone-quality conditions.

Bone grouping	Elastic modulus (MPa)	PFNA length	Cortical bone peak stress (MPa)
A	588	170 mm	185.250
		240 mm	85.191
		320 mm	128.180
B	445	170 mm	189.290
		240 mm	86.359
		320 mm	129.880
C	260	170 mm	195.920
		240 mm	88.161
		320 mm	154.810
D	115	170 mm	203.800
		240 mm	90.125
		320 mm	253.290
E	63	170 mm	208.250
		240 mm	91.167
		320 mm	349.520
F	34	170 mm	211.700
		240 mm	91.948
		320 mm	472.820

Bone groups A–F represent cancellous bone elastic moduli of 588, 445, 260, 115, 63, and 34 MPa, respectively. PFNA, proximal femoral nail antirotation.

### 3.3 Cancellous bone stress analysis

**FIGURE 5:**
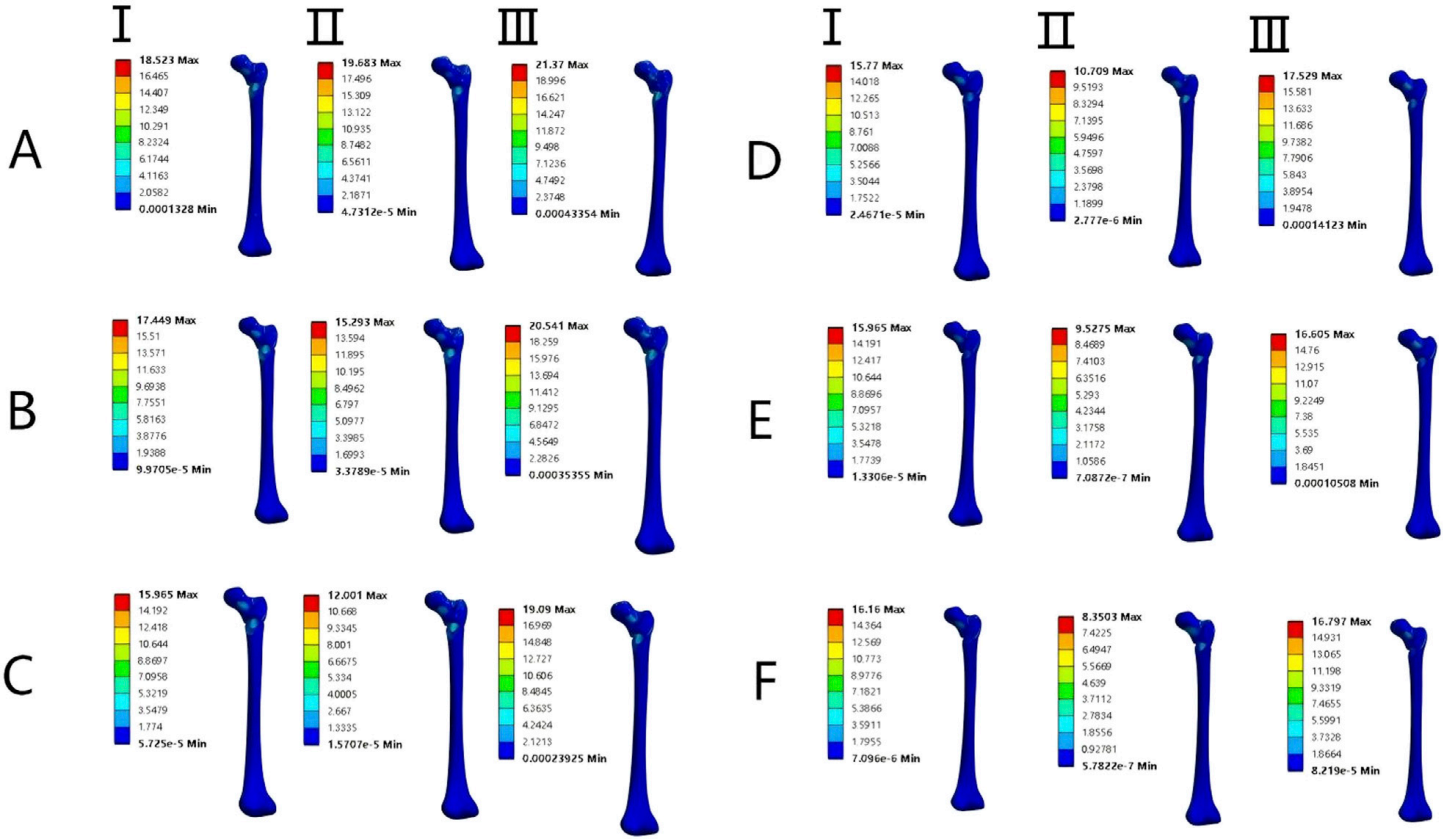
Von Mises stress distribution in cancellous bone under six bone quality conditions with different PFNA lengths. Rows correspond to bone groups **(A–F)**, representing cancellous bone elastic moduli of 588, 445, 260, 115, 63, and 34 MPa, respectively. Columns correspond to PFNA lengths of 170 mm (I), 240 mm (II), and 320 mm (III). Stress values are expressed in MPa, with blue indicating lower stress and red indicating higher stress. The displayed cancellous region mainly includes the femoral head and neck. For grayscale printing, readers are advised to use the numerical labels (Min to Max) provided along the color bar for accurate value quantification.

The von Mises stress distribution contour plots for cancellous bone (Figure 5) revealed that the stress levels for the 240 mm medium-length nail decreased as bone quality deteriorated. The stress values decreased from 19.683 MPa at 588 MPa–8.3503 MPa at 34 MPa, representing a reduction of 57.6%. This stress-decreasing characteristic helps to protect the inherently fragile structure of osteoporotic cancellous bone and reduce the risk of postoperative intrafemoral bone resorption or cut-out. The cancellous bone stress for the 170 mm short nail ranged from 15.77 to 18.523 MPa and exceeded that of the 240 mm nail under poorer bone quality conditions. The cancellous bone stress of the 320 mm long nail ranged from 16.605 to 21.37 MPa, consistently higher than that of the 240 mm nail across all conditions. Even with extremely poor bone quality (34 MPa), it reached 16.797 MPa, which is approximately twice that of the 240 mm nail. The peak von Mises stress in cancellous bone for PFNA implants of different lengths under various bone quality conditions is presented in Table 4.

**TABLE 4:** Peak von Mises stress in cancellous bone under different bone-quality conditions.

Bone grouping	Elastic modulus (MPa)	PFNA length	Cancellous bone peak stress (MPa)
A	588	170 mm	18.5230
		240 mm	19.6830
		320 mm	21.3700
B	445	170 mm	17.4490
		240 mm	15.2930
		320 mm	20.5410
C	260	170 mm	15.9650
		240 mm	12.0010
		320 mm	19.0900
D	115	170 mm	15.7700
		240 mm	10.7090
		320 mm	17.5290
E	63	170 mm	15.9650
		240 mm	9.5275
		320 mm	16.6050
F	34	170 mm	16.1600
		240 mm	8.3503
		320 mm	16.7970

Bone groups A–F represent cancellous bone elastic moduli of 588, 445, 260, 115, 63, and 34 MPa, respectively. PFNA, proximal femoral nail antirotation.

### 3.4 Femoral displacement analysis

**FIGURE 6:**
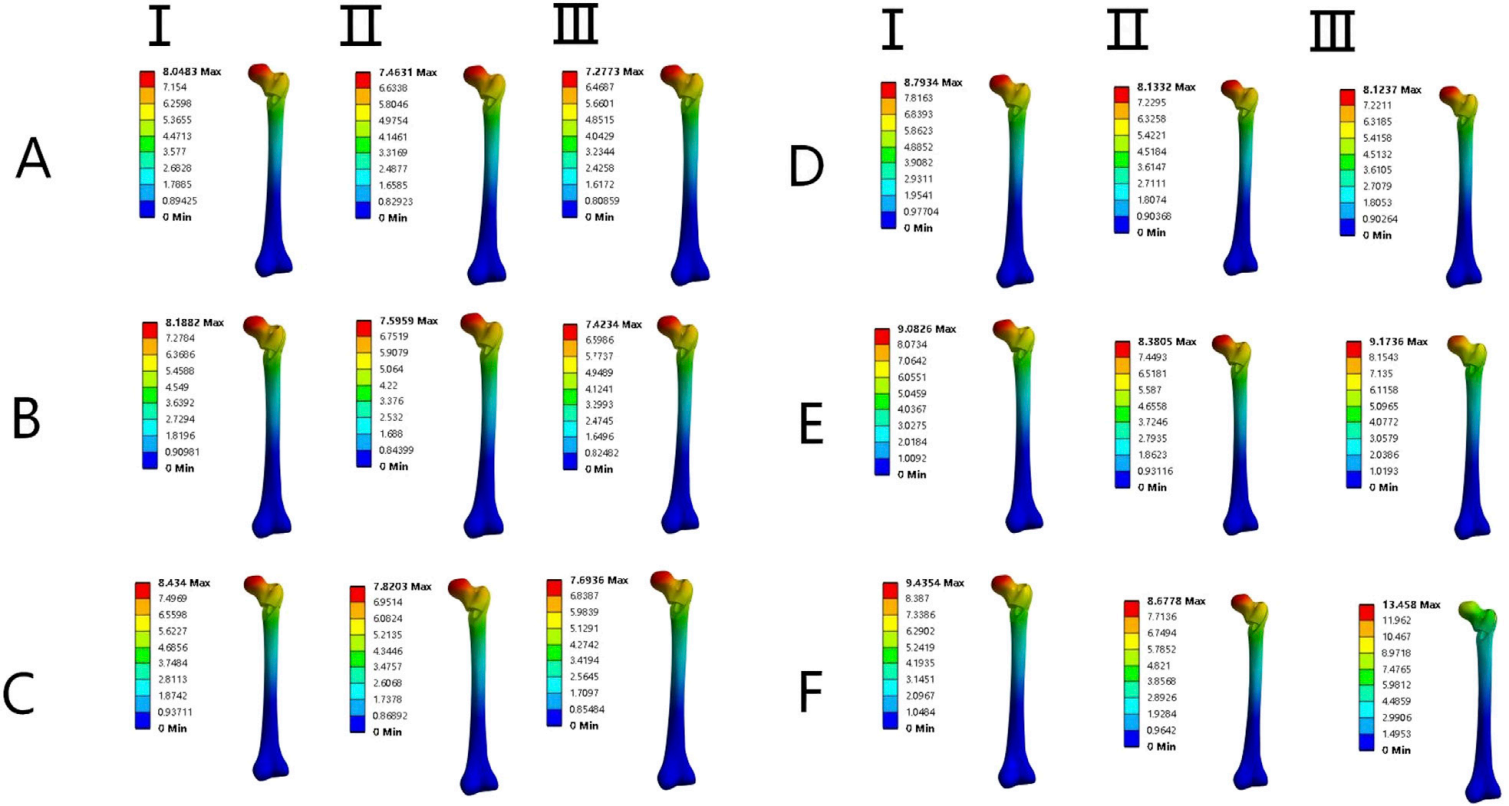
Femoral displacement distribution under six bone quality conditions with different PFNA lengths. Rows correspond to bone groups **(A–F)**, representing cancellous bone elastic moduli of 588, 445, 260, 115, 63, and 34 MPa, respectively. Columns correspond to PFNA lengths of 170 mm (I), 240 mm (II), and 320 mm (III). Displacement values are expressed in mm, with blue indicating lower displacement and red indicating higher displacement. Maximum displacement was observed at the femoral head in all models. For grayscale printing, readers are advised to use the numerical labels (Min to Max) provided along the color bar for accurate value quantification.

All three intramedullary nail groups exhibited maximum femoral displacement in the femoral head load-bearing region (Figure 6). Under fixation with a 170 mm short nail, the femur exhibited maximum displacement values ranging from 8.0483 to 9.4354 mm. This femoral displacement gradually increased with deteriorating bone quality, showing a marked increase when the bone strength decreased below 260 MPa. This indicates poor deformation resistance under osteoporotic conditions, in which micromotion at the fracture site may impair healing. The femoral displacement patterns under fixation with the 240 mm medium-length nail and the 320 mm long nail were similar, with maximum femoral displacements of 7.4631–8.6778 mm and 7.2773–13.458 mm, respectively. Similar numerical values in Groups A-D indicate comparable performances in controlling fragment displacement under mild-to-moderate osteoporosis, whereas the 320 mm nail showed markedly increased displacement in severe osteoporosis (Groups E-F). The maximum femoral displacement values of PFNA fracture models of different lengths under various bone quality conditions are listed in Table 5.

**TABLE 5:** Maximum femoral displacement under different bone-quality conditions.

Bone grouping	Elastic modulus (MPa)	PFNA length	Femoral displacement (mm)
A	588	170 mm	8.0483
​	​	240 mm	7.4631
​	​	320 mm	7.2773
B	445	170 mm	8.1882
​	​	240 mm	7.5959
​	​	320 mm	7.4234
C	260	170 mm	8.4340
​	​	240 mm	7.8203
​	​	320 mm	7.6936
D	115	170 mm	8.7934
​	​	240 mm	8.1332
​	​	320 mm	8.1237
E	63	170 mm	9.0826
​	​	240 mm	8.3805
​	​	320 mm	9.1736
F	34	170 mm	9.4354
​	​	240 mm	8.6778
​	​	320 mm	13.458

Bone groups A–F represent cancellous bone elastic moduli of 588, 445, 260, 115, 63, and 34 MPa, respectively. PFNA, proximal femoral nail antirotation.

### 3.5 Biomechanical performance of 170 mm PFNA with different diameters

Since the primary analysis identified the 170 mm short PFNA as the configuration with the highest implant stress and the least favorable overall biomechanical profile among the tested nail lengths, subsequent diameter analysis was restricted to this construct. This supplementary analysis was designed to determine whether increasing nail diameter could partially offset the mechanical disadvantage of the short nail when its use is necessitated by anatomical constraints, such as excessive femoral anterior bowing, a narrow or deformed medullary canal, or pre-existing ipsilateral distal femoral implants. Accordingly, three diameters (9.5, 10, and 11 mm) were evaluated across the six simulated bone-quality conditions. The 10 mm data were obtained from the original finite element analysis described in Section 3.1. The 9.5 mm and 11 mm models were established with identical loading and boundary conditions as described in Section 2.3, and independently solved.

#### 3.5.1 Implant stress in the 170 mm PFNA with different diameters

The von Mises stress distribution contour plots of the 170 mm PFNA implants across the three diameters demonstrated that stress concentration consistently occurred at the distal locking screw-main nail junction for all diameters, identical to the pattern observed in Section 3.1.

The 9.5 mm nail exhibited peak stresses ranging from 1268.30 to 1295.90 MPa across the six bone quality conditions, exceeding the yield strength of titanium alloy (approximately 900 MPa) in all groups by a considerable margin. Notably, the 9.5 mm nail stress remained relatively constant regardless of bone quality deterioration, varying by only 2.2% between Group A (1268.30 MPa) and Group F (1295.90 MPa). This indicates that at this small diameter, the implant itself becomes the structurally limiting component—the nail is so slender that it bears the majority of the load irrespective of the surrounding bone condition, and the stress magnitude is primarily determined by the implant geometry rather than by bone quality.

The 10 mm nail showed peak stresses of 1054.20–1357.50 MPa, exceeding the yield threshold across all six bone quality conditions, with a clear trend of increasing stress as bone quality deteriorated.

The 11 mm nail demonstrated substantially lower stresses, ranging from 648.76 to 961.96 MPa. In Groups A through E (bone quality ≥63 MPa), the 11 mm nail stresses remained below the yield threshold (648.76–730.22 MPa). Only in Group F (34 MPa), representing extreme osteoporosis, did the 11 mm nail approach and marginally exceed the yield limit (961.96 MPa). Increasing the diameter from 9.5 mm to 11 mm reduced peak implant stress by 25.8%–49.0% depending on bone quality, with the greatest absolute and relative reduction observed under better bone quality conditions (Table 6).

**TABLE 6:** Peak von Mises stress in 170-mm PFNA implants of different diameters under different bone-quality conditions.

Bone group	Elastic modulus (MPa)	9.5 mm	10 mm	11 mm
A	588	1268.3	1054.2	648.76
B	445	1270.7	1079.8	654.16
C	260	1273.6	1130.3	662.16
D	115	1280.2	1215.8	670.51
E	63	1287.6	1286.0	730.22
F	34	1295.9	1357.5	961.96

Bone groups A–F represent cancellous bone elastic moduli of 588, 445, 260, 115, 63, and 34 MPa, respectively. PFNA, proximal femoral nail antirotation.

The peak von Mises stress of 170 mm PFNA implants of different diameters under varying bone quality conditions is presented in Table 6.

## 4 Discussion

The principal finding of this study was that the 240 mm PFNA demonstrated the most favorable overall biomechanical performance across different simulated osteoporosis severities. Compared with the 170 mm configuration, the medium-length nail markedly reduced implant stress and cortical bone stress, indicating a lower risk of stress concentration and mechanical overload. Compared with the 320 mm configuration, it achieved similar displacement with lower peak stress, suggesting that increasing nail length beyond 240 mm did not provide additional biomechanical benefit under the present conditions. Collectively, these findings indicate that the 240 mm PFNA may provide a more balanced mechanical environment for unstable osteoporotic A2.3 intertrochanteric fractures.

From a structural perspective, Osteoporosis is a significant contributing factor to intertrochanteric A2.3 fractures, compromising the quality of internal fixation and potentially leading to secondary fracture displacement due to poor load-bearing capacity (Zhang et al., 2018). The functional morphology of the proximal femoral trabecular system-particularly the orientation and density of primary compressive, secondary compressive, and tensile trabeculae-plays a critical role in load transmission and fracture resistance. Shah et al. provided a detailed anatomical characterization of this trabecular network using P45 sectional plastination combined with 3D reconstruction finite element analysis, demonstrating how regional variations in trabecular architecture influence stress distribution patterns in the proximal femur (Shah et al., 2025). These anatomical insights directly inform the biomechanical rationale for PFNA fixation in osteoporotic bone.

From the perspective of implant safety, stress distribution patterns reveal critical risk points. The 170 mm short nail exhibited peak stresses of 1054.20–1357.50 MPa across the six bone quality conditions, with stress reaching 1130.30 MPa when bone quality decreased to 260 MPa. This dangerous concentration at the distal locking screw hole—a geometric weak point—constitutes a clear risk for fatigue fracture under conditions of diminished bone support. In contrast, both the 240 mm medium-length nail and the 320 mm long nail transferred the primary stress to the structurally more robust spiral blade-main nail junction, significantly enhancing the biomechanical stability. Notably, the 240 mm medium-length nail exhibited the lowest stresses across all six bone quality conditions (442.86–560.44 MPa) with the most gradual increase. Our results are broadly in agreement with those of Hong et al. (2017), who found that, in unstable intertrochanteric fractures, 240-mm and 280-mm PFNA constructs reduced femoral medial stress peaks compared with a shorter 200-mm nail, whereas the biomechanical stability of the 240-mm nail was similar to that of the 280-mm nail. This mechanical advantage may help reduce the stress burden on the implant and could be beneficial for construct durability under diminished bone support. In addition to reducing the mechanical burden on the implant, this stress pattern may also help protect fragile bone tissue. Treatment of osteoporotic fractures must achieve stability while rigorously avoiding iatrogenic bone damage (Yu et al., 2016). Our data indicate that the 170 mm short nail causes significant stress concentration on the distal femoral cortical bone (185.25–211.70 MPa), providing a mechanical explanation for postoperative thigh pain and long-term loosening. In contrast, the 240 mm medium-length nail demonstrated superior stress dispersion capabilities, maintaining cortical bone stress consistently between 85.19 and 91.95 MPa across all six bone quality conditions—nearly unaffected by bone quality variation—thereby suggesting a more protective stress distribution pattern in the surrounding cortical bone. A notable finding is that the 320 mm long nail showed cortical bone stresses comparable to the 240 mm nail under better bone quality (≥260 MPa, 128.18–154.81 MPa), but increased sharply when the elastic modulus fell below 115 MPa, reaching 472.82 MPa at 34 MPa—more than five times that of the 240 mm nail. Similarly, a clinical study by Cha et al. (2024) concluded that changes in nail length showed no significant correlation with femoral head varus deformity and were not an independent risk factor for this complication, suggesting that commonly used intramedullary nails should adequately meet fixation needs without requiring longer nails. This suggests that simply increasing the nail length may not yield sustained benefits and may actually increase the risk of iatrogenic injury in patients with extremely poor bone quality. Within the femoral head cancellous bone, the 240 mm medium-length nail achieved a particularly noteworthy “stress-decreasing” characteristic: as bone quality deteriorated, cancellous bone stress progressively declined from 19.68 to 8.35 MPa, a 57.6% reduction. This characteristic may help prevent spiral blade cut-out and preserve residual bone structure.

Regarding femoral stability, PFNA implants of different lengths demonstrated significant differences in controlling femoral displacement, which was directly related to the initial mechanical environment for fracture healing. The 170 mm short nail maintained relative stability in good bone quality (8.05 mm at 588 MPa) but exhibited rapid femoral displacement increase as bone quality deteriorated, reaching 9.44 mm at 34 MPa. This reveals inadequate control over micromotion, posing a risk of nonunion or malunion. Notably, the 240 mm medium-length nail and 320 mm long nail exhibited similar femoral displacement values under mild-to-moderate osteoporosis (Groups A-D: 7.46–8.13 mm vs. 7.28–8.12 mm), but diverged in severe osteoporosis, where the 320 mm nail showed a pronounced increase (Groups E-F: 9.17–13.46 mm) while the 240 mm nail remained relatively stable (8.38–8.68 mm). However, this difference should be interpreted cautiously, because all models used a single distal locking screw. As longer nails are often fixed with two distal locking screws in clinical practice, part of the increased displacement in the 320 mm model may be related to the simplified distal locking configuration rather than nail length alone. Therefore, within the present modeling conditions, extending PFNA length beyond 240 mm did not demonstrate additional benefit in displacement control. Thus, the 240 mm medium-length nail appeared to provide an adequate biomechanical profile in the present computational setting.

Previous clinical studies provide a practical context for interpreting the potential relevance of the 240-mm medium-length nail (Shannon et al., 2019; Boone et al., 2014). In a randomized prospective trial, Shannon et al. (2019) found comparable functional outcomes and complication rates between short and long cephalomedullary nails. Boone et al. (2014) reported that, compared with long PFNA, short PFNA was associated with simplified procedures, shorter operative time, lower transfusion rates, and less intraoperative blood loss in intertrochanteric femur fractures. These considerations are particularly relevant in elderly patients with osteoporotic fractures, who often present with multiple comorbidities and limited physiological reserve. Although the present finite element study did not evaluate operative time, blood loss, or postoperative outcomes, our biomechanical results are consistent with the possibility that a 240-mm nail may offer a reasonable compromise between mechanical stability and surgical invasiveness.

When a 170 mm short nail is clinically unavoidable because of anatomical constraints, our results suggest that nail diameter should be interpreted in terms of a mechanical threshold rather than a simple linear benefit. The 9.5 mm nail performed poorly under all simulated bone quality conditions, with peak implant stresses of 1268.30–1295.90 MPa, consistently exceeding the yield strength of titanium alloy. This finding indicates that a 9.5 mm nail may not provide sufficient structural resistance to avoid implant overloading and fatigue-related failure, particularly under worst-case loading conditions. Notably, the stress in the 9.5 mm nail changed by only 2.2% between Groups A and F, suggesting that at this small diameter, the implant geometry itself becomes the principal limiting factor, largely independent of the surrounding bone quality. By contrast, increasing the diameter to 11 mm reduced peak implant stress by 25.8%–49.0% and kept the implant stress below the material yield threshold in Groups A–E, with only a slight exceedance in the most severe osteoporotic condition (Group F, 961.96 MPa). These results indicate that increasing nail diameter from 9.5 mm to 10–11 mm substantially improves the mechanical safety margin of the construct. However, the difference between 10 mm and 11 mm requires more cautious interpretation. Although the 11 mm nail showed lower implant stress in the finite element analysis, this advantage may primarily reflect improved resistance to implant fatigue failure rather than a clinically detectable reduction in all types of fixation failure. This distinction may explain why our biomechanical findings differ in emphasis from prior clinical studies. Rinehart et al. (2021) found no significant difference in reoperation rates between 10 mm cephalomedullary nails and nails larger than 10 mm in geriatric intertrochanteric fractures. Likewise, Ergişi et al. (2022) reported no significant difference in implant failure rates between nails ≤10 mm and >10 mm, and Choi et al. (2024) found that the nail–canal diameter ratio did not significantly affect union time or clinical outcomes in patients treated with short cephalomedullary nails. These clinical observations suggest that once nail diameter reaches a mechanically acceptable range, further increases may not translate into measurable improvements in overall clinical outcomes. A likely explanation is that the minimum nail diameter required to prevent implant fatigue failure under worst-case mechanical loading may be lower than the diameter required to prevent other clinically relevant failure modes, such as cut-out, loss of reduction, delayed union, or nonunion. Those outcomes are strongly influenced by multiple additional factors, including fracture reduction quality, tip–apex distance, implant position within the femoral head, bone quality, and postoperative loading. Therefore, although the 11 mm nail provides a biomechanical advantage over the 10 mm nail in terms of lower implant stress, this difference may be clinically less important when other failure mechanisms dominate. In contrast, the 9.5 mm nail appears to fall below a biomechanically acceptable threshold and should therefore be avoided, especially in osteoporotic bone. In summary, our finite element analysis indicates that for the specific scenario in which a 170 mm short nail must be used, nail diameter has its greatest practical importance in avoiding undersizing. The 9.5 mm nail was mechanically unfavorable across all bone quality conditions, whereas 10–11 mm nails provided a more acceptable safety margin, with 11 mm showing the lowest implant stress. Thus, the clinical implication of our findings is not necessarily that 11 mm nails will always produce superior postoperative outcomes compared with 10 mm nails, but rather that nails smaller than 10 mm may expose the construct to an unacceptably high risk of implant overloading and fatigue failure.

Placed within a broader academic context, these findings provide valuable corroboration for current clinical research trends in intramedullary nail length selection. While some studies support shorter nails for certain proximal femoral fractures, multiple comparative clinical investigations have emphasized the importance of longer lever arms for stress redistribution in unstable fractures involving the subtrochanteric region, such as the A2.3 intertrochanteric fractures examined in this study (Nie et al., 2022). In the present finite element study, the 240 mm medium-length nail appeared to represent a practical biomechanical transition point rather than a precise universal “effective length threshold.” This interpretation was based on the combined trends in implant stress, cortical bone stress, cancellous bone stress, and femoral displacement. Compared with the 170 mm short nail, increasing nail length to 240 mm reduced implant stress, decreased distal cortical stress concentration, and improved displacement control, suggesting a more favorable load-sharing pattern. However, extending the nail length further to 320 mm did not provide consistent additional biomechanical benefit and, under some severely osteoporotic conditions, was associated with increased cortical stress and displacement. These findings suggest a point of diminishing biomechanical returns beyond 240 mm within the assumptions of the present model.

Finally, although our study demonstrated that a 240 mm intramedullary nail adequately meets the requirements for PFNA internal fixation in osteoporotic intertrochanteric A2.3 fractures of varying severity, surgeons should select the most appropriate intramedullary nail length based on individual patient fracture characteristics for different types of osteoporotic intertrochanteric fractures to ensure personalized surgical treatment.

This study has some limitations. First, only static loading under a simulated single-leg stance was analyzed, which cannot fully reproduce the cyclic and multidirectional loading conditions encountered during gait and daily activities. As fracture healing and implant failure are strongly influenced by repeated loading, the present model cannot directly evaluate fatigue behavior or long-term fixation durability. Therefore, the apparent biomechanical advantage of the 240 mm PFNA should be interpreted only under static loading conditions, and reduced stress magnitude should not be considered definitive evidence of superior fatigue resistance. Second, bone tissues were modeled as isotropic, homogeneous, and linearly elastic materials. Although this simplification is common in finite element analysis, it does not fully reflect the anisotropic and heterogeneous nature of real bone, particularly trabecular bone. As a result, the absolute stress magnitude and local stress distribution in cancellous bone may differ from *in vivo* conditions, and the observed stress-decreasing characteristic of the 240 mm nail in cancellous bone should be generalized with caution. In addition, although bone stress was used as a comparative indicator of load transfer and stress concentration in the present study, bone strain may provide more direct insight into the local mechanical stimulus relevant to bone adaptation and fracture healing. Therefore, the absence of a strain-based analysis is a limitation of the present work. Third, contact conditions at the implant–bone interface were simplified, which may influence local stress transfer. Fourth, only a single fracture configuration was evaluated, limiting applicability to other fracture patterns. Fifth, the model was not directly validated against cadaveric, experimental, or clinical data. Future studies should incorporate cyclic loading, anisotropic and patient-specific material properties, more realistic contact definitions, additional fracture configurations, and experimental or clinical validation. Nevertheless, this study still provides a controlled comparative biomechanical analysis of PFNA fixation in simulated osteoporotic A2.3 intertrochanteric fractures.

Within the assumptions of this finite element study, the 240 mm PFNA medium-length nail showed a more favorable overall comparative biomechanical profile than the 170 mm short nail across the simulated osteoporotic conditions, while demonstrating performance comparable to that of the 320 mm long nail in terms of femoral stability. The 240 mm construct was associated with lower implant stress and a more balanced stress distribution in bone under the present loading conditions. When use of a 170 mm short nail is clinically necessary, diameters of 10–11 mm may be biomechanically preferable, whereas the 9.5 mm nail appeared mechanically less favorable in osteoporotic bone. These findings suggest that the 240 mm PFNA may represent a reasonable biomechanical option for osteoporotic intertrochanteric A2.3 fractures, although further experimental and clinical validation is required.

## Key messages

- In the present model, the 240 mm PFNA medium-length nail showed a more favorable biomechanical profile than the 170 mm short nail and a stability pattern comparable to that of the 320 mm long nail.
- The 240 mm nail was associated with improved stress distribution and reduced stress concentration under the simulated osteoporotic conditions.
- The 240 mm nail may represent a biomechanically appropriate fixation option under the present model conditions.
- When a 170 mm short nail is clinically necessary, diameters of 10–11 mm may be biomechanically preferable, whereas the 9.5 mm nail appeared mechanically less favorable in osteoporotic bone.

## Strengths and limitations of this study

- Six cancellous bone elastic moduli were used to simulate different osteoporosis severities.
- Multiple biomechanical parameters were analyzed under consistent loading conditions.
- Limitations include static loading, simplified bone properties, and a single fracture model.

## Data Availability

The original contributions presented in the study are included in the article/supplementary material, further inquiries can be directed to the corresponding author.
